# Development of a Deep-Learning Model for Automated Detection and Quantification of Bleeding in Unilateral Biportal Endoscopic Spine Surgery

**DOI:** 10.3390/jcm15051934

**Published:** 2026-03-04

**Authors:** Takaki Yoshimizu, Daisuke Sakai, Daiki Morita, Meng-Huang Wu, Teruaki Miyake, Sanshiro Saito, Tetsutaro Mizuno, Ushio Nosaka, Keisuke Ishii, Mizuki Watanabe, Kanji Sasaki

**Affiliations:** 1 Department of Orthopaedic Surgery, Seirei Hamamatsu General Hospital, Hamamatsu 430-8558, Japan; 2 Department of Orthopaedic Surgery, Tokai University Hospital, Isehara 259-1193, Japan; daisakai@tokai.ac.jp; 3 Department of Plastic and Reconstructive Surgery, Tokai University Hospital, Isehara 259-1193, Japan; d-morita@tokai.ac.jp; 4 Department of Orthopedics, Taipei Medical University Hospital, Taipei 110, Taiwan; 5 Department of Spine and Bone Tumors, Seirei Hamamatsu General Hospital, Hamamatsu 430-8558, Japan; 6 APSS-AOSpine Frontier Technologies Research Group

**Keywords:** endoscopy, spine, hemorrhage, deep learning, artificial intelligence, computer-assisted

## Abstract

**Objectives:** To develop and validate a deep-learning model capable of detecting and quantifying intraoperative bleeding to objectively evaluate visual field impairment in unilateral biportal endoscopic spine surgery (UBE). **Methods:** Overall, 223,568 still images were extracted from 20 UBE videos and used to train a U-Net++ segmentation model based on the red masks generated using hue, saturation, and value (HSV) thresholding. The model was fine-tuned using 350 manually annotated images that differentiated clinically relevant bleeding (red masks) from non-bleeding red regions (zero masks). The model performance was evaluated against 180 ground-truth images annotated by three spine surgeons, which were extracted from videos that were separate from those used for training and fine-tuning. Dice and intersection-over-union (IoU) scores were calculated, and correlation analyses were performed based on inter-annotator agreement. **Results:** The HSV-based model reproduced the red regions with high fidelity; however, it showed limited agreement with the ground-truth bleeding regions (median Dice = 0.57, IoU = 0.40). The fine-tuned model improved substantially. For image-wise binary classification of bleeding presence, the model achieved an accuracy of 86%, with a sensitivity of 93% and a specificity of 60%. For pixel-level segmentation performance, the model achieved a median Dice score of 0.79 and a median IoU of 0.65 on ground-truth-positive images. Dice performance exceeded 0.80 in cases with strong inter-surgeon ground-truth concordance (≥0.80) and substantial bleeding area (>20%). **Conclusions:** This deep-learning model can accurately detect clinically meaningful intraoperative bleeding in UBE and quantify visual field impairments in still images and surgical videos. Future applications include the evaluation of hemostatic techniques, postoperative image-based assessment of surgical quality, and real-time intraoperative bleeding alerts to support surgical decision-making.

## 1. Introduction

Unilateral biportal endoscopic spine surgery (UBE) has emerged as an innovative minimally invasive technique that provides a wide surgical field and excellent maneuverability, allowing surgeons to perform decompression and discectomy through two independent portals under continuous irrigation. As its clinical indications continue to expand, UBE is expected to become a standardized procedure in spine surgery [1].

However, because UBE relies on continuous irrigation and a highly magnified view, even minor bleeding can rapidly obscure visualization. Intraoperatively, this visual obstruction not only increases the risk of dural tears and conversion to open surgery but also leads to prolonged operative times, increased surgeon workload, and the need for higher irrigation pressures. Postoperatively, subjective evaluation of hemostasis within the limited endoscopic field may leave small, persistent bleeders unnoticed, potentially resulting in spinal epidural hematomas. The hemostatic strategy in UBE has not yet been standardized, and variations in irrigation flow, pressure control, and use of hemostatic devices may further influence intraoperative visibility.

In spinal surgery in general, the incidence of symptomatic postoperative spinal epidural hematoma has been reported to be approximately 0.5%, with minimally invasive approaches showing slightly higher rates than conventional techniques [2]. Recent systematic reviews of UBE have reported postoperative epidural hematoma and dural tear rates ranging from 0.27% to 1% and 2% to 2.5%, respectively [3,4,5]. Although these complications are not directly caused by intraoperative bleeding, maintaining a clear visual field through adequate hemostasis is critical for procedural safety. Nevertheless, objective and standardized methods for evaluating intraoperative bleeding are lacking, leaving surgeons to rely on subjective visual judgment.

Recently, artificial intelligence (AI) has shown promising applications in surgical planning, imaging analysis, and intraoperative guidance. Its adoption has rapidly increased in parallel with the refinement of minimally invasive spine surgery techniques. AI has the potential to support personalized surgical decision-making and enhance intraoperative safety, although this approach remains in its early stages [6,7]. In gastrointestinal endoscopy, AI-based systems have enabled real-time bleeding detection and lesion identification [8]. However, despite these advances, AI has not yet been applied to bleeding detection or visual field assessment in spinal endoscopy. This gap highlights the novelty and clinical importance of the present study [9].

This pilot study aimed to develop and validate a deep-learning model as an initial approach for the objective detection and quantification of bleeding in spinal endoscopy. By exploring the feasibility and utility of a reliable means of assessing intraoperative bleeding, we sought to provide foundational evidence for future strategies to evaluate hemostatic control and to enhance the safety and effectiveness of UBE procedures.

## 2. Materials and Methods

### 2.1. Ethics Statement

This retrospective study was approved by the Institutional Review Board of our institution. Written informed consent for the use of surgical videos for research purposes was obtained from all participating patients.

### 2.2. Deep Learning Workflow Design

**Figure 1:**
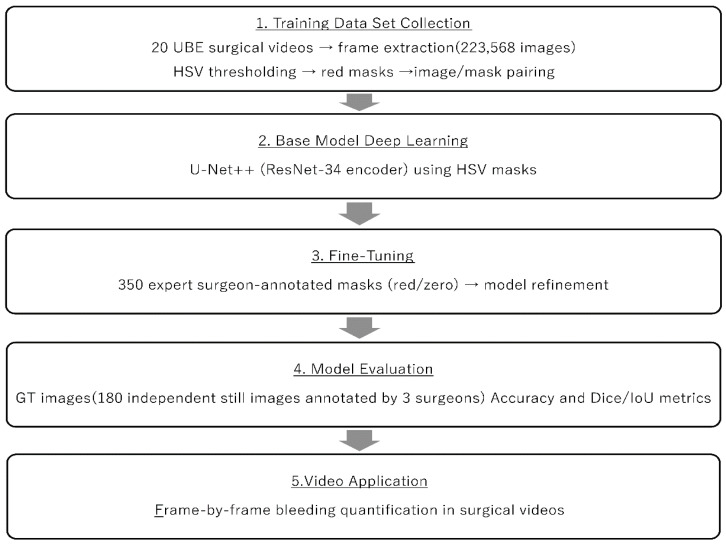
Deep learning workflow design. A workflow was designed in which a base model was created using a large dataset of endoscopic images masked by red pixels, followed by fine-tuning to learn clinically meaningful bleeding.

This study was designed based on the hypothesis that a two-step deep learning workflow improves the precision and clinical relevance of bleeding detection (Figure 1). In the first step, a base model was trained using a large-scale dataset of endoscopic images paired with hue, saturation, and value (HSV)-based masks. This extensive training allowed the network to comprehensively learn the unique visual patterns of the endoscopic field and automatically extract all red regions, achieving more detailed and consistent segmentation than manual annotation alone. In the second step, the base model was fine-tuned using a highly curated dataset, in which UBE expert manually excluded clinically irrelevant red areas, such as bone marrow and vasculature. The model’s performance was validated by comparing its outputs with ground-truth images manually annotated by experienced surgeons. Finally, we confirmed the model’s feasibility for video-level assessment by performing frame-by-frame inference on recorded surgical videos and reconstructing them into a processed output.

### 2.3. Training Dataset Collection

In this study, 20 videos of UBE procedures comprising discectomy, decompression, and fusion surgeries performed at a single institution by multiple surgeons were used to construct a training dataset. Endoscopic videos were recorded using either full high-definition or 4K image storage systems. Cases with specific intraoperative complications or poor video quality were excluded to ensure the dataset was reliable. From these videos, 223,568 original resolution images were extracted at a rate of 2 fps. Each extracted image was subsequently converted to the HSV color space, and a corresponding binary mask was generated to extract red regions within the endoscopic field.

The HSV threshold parameters were defined by a surgeon with experience in >300 UBE procedures, encompassing regions that could clinically be perceived as red, such as bleeding, vasculature, and cancellous bone. Specifically, the conditions were as follows: hue, 0–8°; 172–180°; saturation, 90–255; and value, 50–255. Concurrently, a field-of-view mask (saturation > 60, value > 70) was applied to explicitly exclude the lateral black margins of the endoscope from the region of interest, thereby eliminating meaningless background. The resulting masks were saved as 8-bit grayscale images, with the red regions represented in white.

Subsequently, the original images and the masks were resized to 512 × 512 pixels. Only images with a red area ratio exceeding 1% within the endoscopic field were selected as training data to exclude insignificant red noise. Overall, 145,763 image–masks pairs containing clinically meaningful red regions were used to train the deep learning model.

### 2.4. Base Model for Deep Learning

The U-Net++ architecture, which is specialized for image segmentation, with ResNet-34 as the encoder, was employed in the deep-learning model used in this study. All layers were randomly initialized without using any pretrained weights, such as those from ImageNet. The training labels comprised red mask images generated using the aforementioned HSV thresholding conditions.

All input images were in the RGB format and resized to 512 × 512 pixels. The pixel values were scaled to a range of 0–1 and subsequently standardized using a channel-wise mean of [0.485, 0.456, 0.406] and a standard deviation of [0.229, 0.224, 0.225] to stabilize the training process.

Binary cross-entropy with logit loss was used as the loss function, and the Adam optimizer was applied at a learning rate of 1 × 10^−4^. The batch size was set to 4, and early stopping with a patience of 20 epochs was used to prevent overfitting.

### 2.5. Fine-Tuning Conditions

The base model, trained using HSV-based masks, was designed to indiscriminately extract all the red regions, including those with limited clinical relevance, such as vascular redness or cancellous bone. Therefore, fine-tuning was performed to specialize the model to detect clinically meaningful bleeding.

The fine-tuning dataset comprised 350 highly curated images selected from the first-stage training pool derived from the 20 surgeries used to train the base model. Rather than relying on random sampling, an experienced UBE surgeon purposively selected these frames across various surgical phases (e.g., bone drilling, soft tissue decompression) to ensure maximum morphological diversity. Annotations were meticulously delineated using precise polygon approximations via Labelme software (v5.6.0), which were subsequently converted into pixel-wise binary masks. Specifically, 120 red mask images representing definite bleeding and 230 zero-mask images representing non-bleeding red areas, such as vessels or cancellous bone, were used.

The model architecture used for fine-tuning was the same U-Net++ built with a ResNet-34 encoder, and the training was resumed using the weights from the base model. All input images were in the RGB format and were resized to 512 × 512 pixels. Data augmentation techniques such as horizontal flipping and variations in brightness and saturation were applied.

Binary cross-entropy with logit loss was used as the loss function, and the model was optimized using the Adam optimizer with a learning rate of 1 × 10^−4^. However, early stopping was not based on loss values; instead, it was implemented using the Dice coefficient and IoU scores calculated against the ground-truth (GT) masks (described later). The training was terminated if no improvement in these metrics was observed for 10 consecutive epochs.

### 2.6. Model Evaluation

To prevent any risk of data leakage, the evaluation dataset was constructed from three UBE surgeries (lumbar disk herniation, spinal canal decompression, and foraminal decompression) that were strictly independent of the training and fine-tuning datasets. Specifically, these evaluation cases were completely excluded at the patient and video levels from all training phases. Although the evaluation surgeries were performed by the same primary surgeon included in the training dataset, the videos were uniformly recorded under standardized 4K high-definition conditions—with consistent camera systems, recording devices, and lighting—to ensure a rigorous assessment of the model’s true performance.

From these independent cases, 60 images representing a balanced distribution of bleeding-area ratios were extracted. Initially, a panel of five orthopedic surgeons—each with 1 to 5 years of UBE experience and having performed 100 to 300 procedures—independently annotated the bleeding areas to generate GT masks. To ensure the reliability of the reference standard as a reflection of average expert perception, inter-observer agreement was assessed using pairwise Dice and IoU coefficients. Because excessive variation in the ground truth makes it impossible to accurately evaluate an AI model’s true performance, we restricted the final reference panel to the three surgeons who demonstrated the highest mutual consistency. This rigorous selection resulted in a final set of 180 highly reliable GT masks (60 images × 3 surgeons). The average Dice and IoU values among these three selected experts were subsequently used to define the GT agreement.

For the binary detection of bleeding (presence/absence), we evaluated image-wise classification performance. The output probability maps from the fine-tuned model were binarized using a sigmoid function and a threshold of 0.5 to generate predicted masks. To exclude regions outside the surgical field, circular field-of-view masks were applied to both the GT and predicted masks. Based on these binarized outputs, we reported overall accuracy, sensitivity, specificity, positive predictive value (PPV), and negative predictive value (NPV), along with the confusion matrix.

For area-based segmentation performance, we focused on the overlap between the predicted and GT masks. Dice and IoU were calculated strictly on GT-positive images (GT bleeding area > 0) to avoid artificial overestimation from true-negative zero-mask cases, in which both GT and predictions were empty. These scores were computed by comparing the predicted masks with each individual surgeon’s GT mask, and the average values were used for evaluation.

To further characterize segmentation performance, we examined how Dice and IoU varied according to GT agreement and bleeding extent. First, the correlations between the model’s Dice/IoU scores and the GT agreement rates were analyzed. Based on the GT agreement rate, images were categorized into three groups (≥0.70, ≥0.80, and ≥0.90), and the model’s performance was summarized within each group. Furthermore, we restricted the area-based subgroup analysis to GT-positive images and categorized them into two groups according to the bleeding-area ratio: a low-bleeding group (>0–20%) and a high-bleeding group (>20%), enabling a comparison of model performance across different levels of bleeding extent.

### 2.7. Video Application

Frame-by-frame inference was performed on recorded UBE surgical videos to evaluate the model’s performance in a dynamic environment. Each frame was resized to 512 × 512 pixels and processed using the fine-tuned model. The bleeding-area ratio was quantified by calculating the proportion of pixels predicted as bleeding relative to the total number of pixels in each frame. For evaluation, composite videos were generated by synchronizing the original endoscopic footage, the model’s segmentation overlay, and a temporal plot representing the calculated bleeding-area ratio over time. Furthermore, to evaluate the feasibility of real-time clinical integration, the model’s computational performance was assessed. The inference speed (frames per second, FPS) and processing latency per frame were measured using 4K-resolution (3840 × 2160 pixels) endoscopic video inputs operating on an NVIDIA GeForce RTX 5070 Ti GPU (Blackwell architecture, 8960 CUDA cores, 16 GB GDDR7 memory).

### 2.8. Statistical Analysis

Statistical analyses were performed using Python software (version 3.8.20; Python Software Foundation). Continuous variables are presented as mean ± standard deviation or median with interquartile range, depending on distribution.

Diagnostic performance metrics including accuracy, sensitivity (recall), specificity, positive predictive value (PPV), and negative predictive value (NPV) were calculated based on the confusion matrix. Confidence intervals (95% CI) for sensitivity, specificity, PPV, and NPV were computed using the Clopper–Pearson exact method.

The assumption of normality for the performance metrics was assessed using the Shapiro–Wilk test. Accordingly, Spearman’s rank correlation coefficients were calculated to evaluate the relationship between the model performance metrics and GT agreement rates. Two-tailed *p*-values were reported, and statistical significance was set at *p* < 0.05.

### 2.9. AI Usage in Model Development

During the implementation of the deep-learning architecture, an artificial intelligence tool (ChatGPT GPT-5; OpenAI, San Francisco, CA, USA) was utilized to assist in generating and refining the source code. All AI-assisted code segments were rigorously reviewed, tested, and validated by the authors to ensure technical accuracy and the integrity of the resulting model.

## 3. Results

### 3.1. Establishment of Ground Truth for Model Evaluation

Initially, the overall average pairwise Dice score across all five surgeons evaluating the 60 test images was 0.66, indicating substantial inter-rater variability. By restricting the final ground-truth consensus panel to the three most consistent experts, the average pairwise Dice score significantly improved to 0.78, with an average IoU of 0.71.

To further characterize this variability, we evaluated the diagnostic performance of each individual surgeon against the consensus ground truth of the top three surgeons. The detailed performance metrics for each surgeon are summarized in Table 1. Notably, the average sensitivity and specificity across all five surgeons were 0.96 and 0.84, respectively. Individual sensitivities ranged from 0.78 to 1.00, while specificities showed a broader variation, ranging from 0.43 to 1.00.

This highly reliable subset of 180 evaluations (60 images × 3 surgeons) was subsequently used as the reference standard to assess the AI model’s performance.

### 3.2. HSV Mask-Based Model

**Figure 2:**
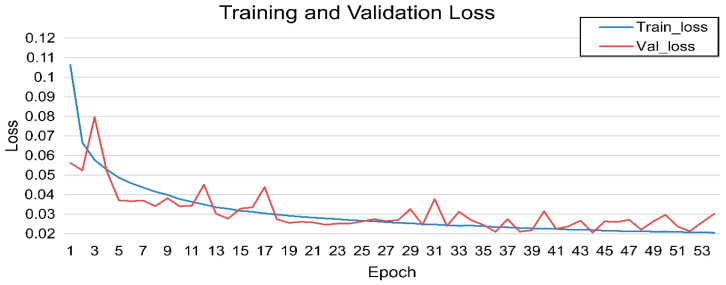
HSV mask-based model: training and validation loss. Model performance improved steadily until epoch 44; however, it showed no further improvement thereafter, and training was terminated at 54 epochs.

The initial model was trained for 54 epochs using automatically generated red masks based on the predefined HSV threshold conditions. The training and validation losses progressively decreased over the epochs, and the validation loss plateaued after epoch 44, at which point early termination was triggered. The best-performing model was obtained at epoch 44, with training and validation losses of 0.022 and 0.021, respectively (Figure 2).

**Figure 3:**
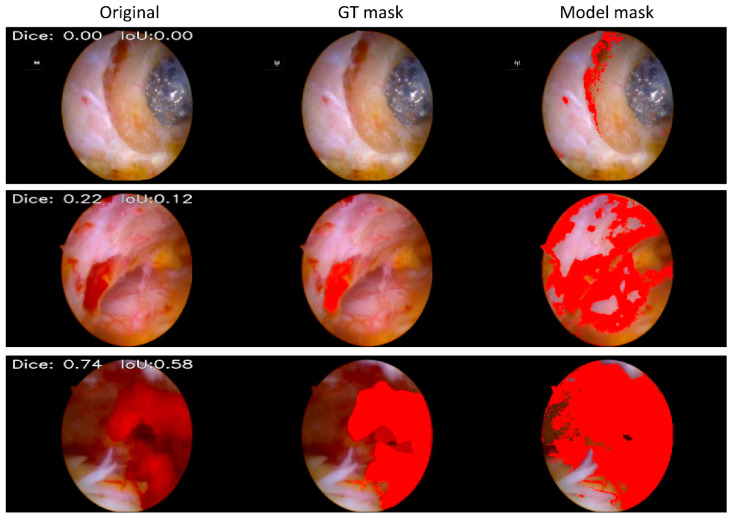
Comparison of HSV mask-based model masks and GT masks. (**Top**): In the cross-section of the resected lamina, cancellous bone was not judged by surgeons as bleeding; nonetheless, it was masked by the HSV mask-based model. (**Middle**): Peridural vessels were not considered bleeding by the surgeons but were masked by the HSV mask-based model. (**Bottom**): Dark red in the deep field, judged by surgeons as not affecting the surgery, was masked by the HSV mask-based model.

On the HSV-based validation masks, the model achieved a Dice coefficient of 0.97 and an IoU of 0.95. When evaluated against expert-annotated GT bleeding masks, the overall average Dice score was 0.24, and the per-surgeon Dice scores were 0.24, 0.22, and 0.25, respectively (Figure 3).

### 3.3. Fine-Tuned Model

The fine-tuned model triggered early stopping at epoch 37, when no further improvement in the validation Dice score was observed for more than 10 consecutive epochs. The best performance was recorded at epoch 27.

Using the best model, the confusion matrix for bleeding detection (presence/absence) across 180 independent evaluations (60 images × 3 surgeons) showed 129 true positives, 25 true negatives, 17 false positives, and 9 false negatives. All nine false-negative images had bleeding occupying <5% of the endoscopic field.

**Figure 4:**
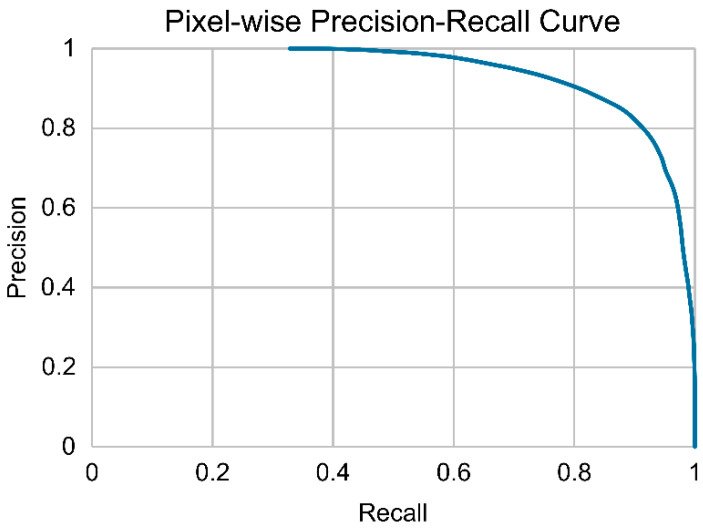
Pixel-wise precision–recall analysis. The graph showed high discriminative performance, with an average precision of 0.94.

The overall image-wise classification accuracy for detecting bleeding was 0.86 (95% CI: 0.80–0.90). The sensitivity, specificity, positive predictive value, and negative predictive value were 0.93 (95% CI: 0.88–0.97), 0.60 (95% CI: 0.44–0.73), 0.88 (95% CI: 0.82–0.93), and 0.74 (95% CI: 0.57–0.85), respectively (Table 2). Pixel-wise precision–recall analysis yielded an average precision of 0.94 (Figure 4).

**Figure 5:**
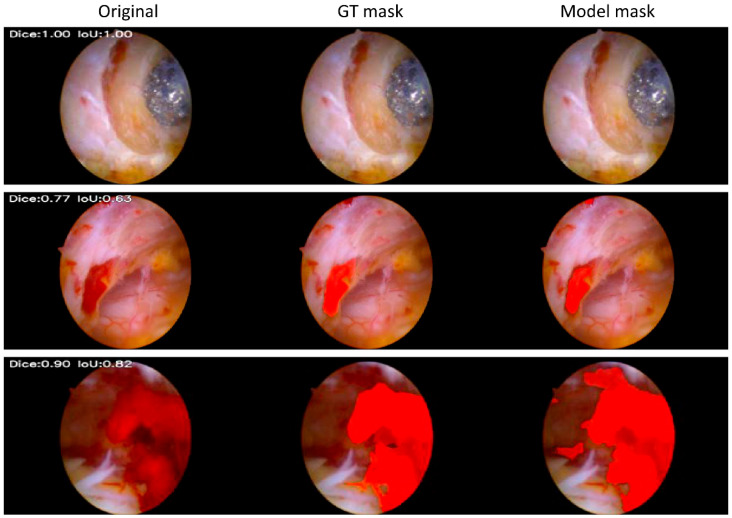
Comparison of fine-tuned model masks and ground truth masks. (**Top**): After fine-tuning, the model no longer identified cancellous bone as bleeding. (**Middle**): After fine-tuning, the model no longer identified the peridural vessels as bleeding. (**Bottom**): After fine-tuning, the model extracts bleeding regions with a more precise morphology.

For area-based segmentation performance on GT-positive images, the overall median Dice and IoU values were 0.79 and 0.65, respectively. The overall mean Dice and IoU values were 0.67 and 0.59, respectively. By surgeon, the median Dice values were 0.81, 0.76, and 0.65, with corresponding median IoU values of 0.68, 0.62, and 0.48. The mean Dice values by surgeon were 0.74, 0.67, and 0.57, with corresponding mean IoU values of 0.63, 0.57, and 0.47, respectively (Table 3 and Figure 5).

**Figure 6:**
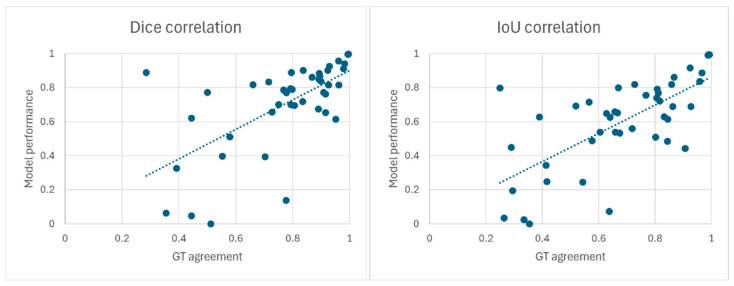
Correlation between ground-truth Dice/IoU agreement rates and model. Dice and IoU values showed strong positive correlations with the inter-surgeon agreement rate of ground-truth masks.

Correlation analysis between GT agreement and model performance in GT-positive images showed Spearman’s rank correlation coefficient of ρ = 0.66 (*p* < 0.001) for Dice and ρ = 0.67 (*p* < 0.001) for IoU (Figure 6).

In the stratified analysis based on GT agreement rates, 34 images with GT agreement ≥ 0.70 showed a median Dice of 0.71 and a median IoU of 0.55. In the subset with GT agreement ≥ 0.80, the median Dice and IoU increased to 0.81 and 0.68, and in those with GT agreement ≥ 0.89 (*n* = 14), they reached 0.89 and 0.80, respectively (Table 4).

For the subgroup analysis according to the GT bleeding-area ratio in GT-positive images, cases were divided into a low-bleeding group (>0–20% of the GT area; *n* = 56) and a high-bleeding group (>20% of the GT area; *n* = 82). The low-bleeding group had a median Dice of 0.53 and a median IoU of 0.36, whereas the high-bleeding group showed higher median Dice and IoU values of 0.83 and 0.71, respectively (Table 5).

### 3.4. Video Application

The application of the fine-tuned model to UBE surgical videos was successfully demonstrated. By comparing the inference masks with the original endoscopic footage, we confirmed the model’s ability to consistently track bleeding regions in a dynamic environment. The bleeding-area ratio within the endoscopic field was quantified frame-by-frame and plotted chronologically, enabling an objective and continuous assessment of bleeding dynamics. Evaluation using the generated composite videos confirmed that the model could effectively visualize the transition of visual field impairment throughout the surgical procedure (Video S1).

Regarding computational performance for real-time clinical integration, the system achieved an average inference speed of 77.92 FPS, with a processing latency of approximately 12.83 ms per frame.

## 4. Discussion

Among the various spinal endoscopic techniques, UBE stands out because it is minimally invasive while providing a broad operative field. Its expanding adoption reflects its potential to become a standard procedure in spine surgery [10,11]. In existing reviews, the incidence of postoperative epidural hematoma reportedly ranges from 0.27% to 1% in lumbar UBE, whereas studies on cervical UBE have described rates of approximately 3% to 5% [4,5,12]. Another important consideration is that, owing to the continuous irrigation setting and magnified endoscopic view, even minor bleeding can swiftly compromise visibility, complicate surgical maneuvers and potentially lead to open conversion [13]. Furthermore, because the procedure is performed under continuous irrigation, the exact amount of intraoperative bleeding cannot be accurately assessed. This limitation may result in surgery proceeding based on subjective assessments that may fail to reflect the true extent of bleeding, potentially leading to unrecognized hemodynamic changes or underestimated blood loss within the irrigated field [14].

Such postoperative hematomas and open conversions caused by impaired endoscopic visualization can ultimately lead to neurological deterioration and prolonged hospitalization, thereby undermining the greatest advantage of endoscopic surgery: its minimally invasive nature. Hemostasis, a fundamental aspect of UBE, is therefore one of the most critical technical elements. Proper intraoperative hemostasis is essential to reduce the occurrence of postoperative hematoma; however, there is currently no established quantitative framework or metric to objectively evaluate hemostatic techniques during surgery.

In this pilot study, we developed and evaluated a deep-learning model capable of quantitatively assessing visual obstruction caused by bleeding during UBE procedures. Bleeding regions during endoscopic surgery are highly dynamic and morphologically irregular. In the surgical field, blood often mixes with tissues and irrigation fluids, resulting in ambiguous boundaries that are difficult to define. Accurate segmentation under these conditions requires a model that can capture fine structural details and spatial variations across frames. We adopted U-Net++, an extension of the standard U-Net architecture that incorporates dense skip connections across multiple nested layers. This design enables more effective integration of low-level spatial information and high-level semantic features, facilitating accurate detection of both diffuse and sharply defined bleeding regions [15]. This capability is particularly beneficial for precise estimation of the bleeding area.

We first trained a base model to detect red areas using HSV thresholds. The ResNet-34 encoder was initialized randomly without conventional ImageNet pretraining. This decision was driven by the substantial domain gap between natural images and the unique structural and visual characteristics of endoscopic surgery. Specifically, endoscopic images possess a distinct geometric layout—a circular surgical field surrounded by a black background—which differs fundamentally from the edge-to-edge spatial distribution of objects in standard natural images. By learning from our endoscopic dataset from scratch, the network successfully acquired a domain-specific foundation optimized for this unique spatial layout as well as the uniquely red-dominated, constantly irrigated environment. The model achieved high Dice scores relative to the HSV masks, indicating strong learning of all red-colored structures, including vessels and cancellous bone. However, the model showed poor agreement with clinically relevant bleeding areas, suggesting that color thresholding alone was insufficient for isolating meaningful bleeding regions. To address this issue, the base model was subsequently fine-tuned using expert-annotated masks that explicitly distinguished clinically meaningful bleeding (red masks) from irrelevant red regions such as vessels, cancellous bone, and reflections (zero masks). Fine-tuning, in this context, refers to a transfer learning approach in which the weights of a pre-trained model are used for initialization, followed by additional training on task-specific data to refine performance for the clinically relevant target.

Although the fine-tuning dataset was relatively small, our preliminary experiments revealed an important finding: simply increasing the number of fine-tuning images did not necessarily improve the model’s agreement with the independent ground truth. During the developmental phase, we empirically tested approximately 10 different dataset configurations. We observed that including overly ambiguous or highly complex images tended to confuse the model and increase the risk of overfitting. Therefore, an experienced surgeon purposively curated a dataset of 350 representative and unambiguous images (120 with definite bleeding, 230 with clear non-bleeding red structures) to provide clear, high-quality educational examples for the model. By prioritizing annotation quality and clear morphological features over sheer volume, combined with robust data augmentation, the moderate capacity of the ResNet-34 encoder, and rigorous early stopping, the model effectively avoided overfitting and generalized well to the independent test set.

The high sensitivity (0.93) demonstrated by the model is directly attributable to our strategy of using a base model trained with HSV thresholds to comprehensively extract all red regions within the endoscopic field. This initial stage of “exhaustive red region extraction” served as the foundation for a “safety-first” design, prioritizing the avoidance of missed bleeding. However, this design led to a moderate specificity of 0.60, highlighting the inherent challenge of distinguishing clinically significant hemorrhage from ambiguous red signals. Our inter-rater analysis (Table 1) underscores this difficulty, revealing that even experienced surgeons vary significantly in their diagnostic thresholds; notably, the model’s performance falls within the range of expert clinical judgment, where some surgeons demonstrate specificities as low as 0.43. This suggests that the model’s current behavior reflects a cautious, high-sensitivity threshold similar to that of some clinicians, prioritizing the detection of potential bleeding over the exclusion of ambiguous signals. Crucially, the model’s detection challenges were primarily confined to minimal bleeding; as noted in our results, all false-negative images were limited to cases where the bleeding area occupied less than 5% of the endoscopic field. While the model still faces challenges in detecting minimal bleeding points, its current performance provides a quantitative baseline that aligns with the protective decision-making process of surgeons. By providing an objective metric for visual obstruction, this study establishes a foundational framework for standardized hemostatic evaluation in UBE.

Regarding the segmentation quality, our two-stage training strategy effectively refined the model’s ability to delineate bleeding contours. In this context, the Dice coefficient and the IoU are widely used overlap-based metrics for evaluating image segmentation performance, quantifying the agreement between predicted and ground-truth masks. Both metrics range from 0 to 1, where 1 indicates perfect overlap and 0 indicates no overlap; Dice can be regarded as a pixel-wise F1-score, whereas IoU is a more conservative overlap measure defined as the ratio between the intersection and the union of the predicted and ground-truth masks [16]. The observed inter-rater variability (initial mean Dice: 0.66) likely reflects the inherent biological ambiguity of endoscopic bleeding, where blood frequently mixes with irrigation fluid, creating indistinct boundaries that pose challenges for manual annotation even among experts. In our study, the final ground truth was defined as the average expert perception of the most consistent raters, providing a robust and clinically realistic reference for meaningful hemorrhage. In our study, the fine-tuned model achieved a median Dice of 0.79 and a median IoU of 0.65 on GT-positive images. Given the inherent complexity of endoscopic bleeding patterns—including continuous irrigation, motion blur, and the presence of vessels and cancellous bone—these values indicate that the model can reproduce clinically relevant bleeding contours with a high level of fidelity. Although these metrics are not directly equivalent to individual inter-surgeon Dice because of the differences in reference standard construction, the model’s performance closely approaches the range of agreement observed among our experienced surgeons (median Dice: 0.78). This high fidelity suggests that the subsequent fine-tuning effectively taught the network to distinguish clinically significant bleeding from irrelevant red signals by focusing on essential morphological features.

The primary objective of this model was to reliably detect and quantify clinically meaningful bleeding during surgery. From this perspective, stratified performance evaluation based on the GT bleeding-area ratio provided additional insights. In GT-positive images with a bleeding-area ratio >20%, the model demonstrated high reproducibility, with median Dice and IoU scores of 0.83 and 0.71. By contrast, in frames with smaller bleeding areas (>0–20%), the Dice and IoU scores were more variable, particularly in cases with <1% bleeding. This discrepancy reflects the inherent difficulty of accurately aligning the precise morphology of subtle hemorrhages, where even a slight pixel-wise deviation between the predicted and ground-truth masks leads to a disproportionately large decrease in overlap-based scores. This pattern is consistent with the profile of false-negative cases and highlights an area for future improvement—namely, enhancing sensitivity and boundary precision for subtle bleeding—while preserving the high specificity demonstrated in this pilot study. Collectively, these findings suggest that the model achieves highly reproducible performance for clearly defined, clinically significant bleeding, especially when there is strong consensus among experienced operators, and can therefore be used as an objective tool for intraoperative bleeding assessment and monitoring visualization quality.

In addition to still-image evaluation, this model can process complete surgical videos, calculate bleeding-area ratios for each frame, and visualize temporal changes, enabling an objective assessment of field clarity over time. Regarding its computational efficiency, the system achieved an average inference speed of 77.92 FPS with a latency of only 12.83 ms per frame when processing 4K video inputs on an NVIDIA GeForce RTX 5070 Ti GPU. This performance substantially exceeds standard video frame rates (30–60 FPS), demonstrating that the current architecture is capable of high-fidelity, real-time bleeding quantification without intraoperative delay. Such video-based applications may support quantitative evaluation of hemostatic techniques or irrigation strategies, contribute to standardized reporting of endoscopic visibility, and provide a basis for training and quality assurance in UBE.

This study had several limitations. First, external validation was not performed using data from other institutions, which limits the generalizability of the model across different surgical settings and equipment. Second, the training and evaluation datasets were derived from a limited number of surgeons, raising the possibility of operator-related bias. Third, our selection of the three most consistent surgeons to define the ground truth may have introduced a reference standard inflation bias. While this ensured high-quality training data for this pilot study, it may not fully capture the ecological validity of the broader, more ambiguous rater disagreements encountered in clinical practice. Fourth, as highlighted by the false-negative cases, the model’s sensitivity to minimal bleeding (<5% area) was suboptimal. While such minute bleeding may not immediately impair visibility under continuous irrigation, missing these “hidden” sources could potentially lead to postoperative complications, such as epidural hematomas, representing a clinically meaningful limitation. Finally, potential hardware and software constraints may affect the feasibility of applying the model to real-time video analysis, particularly in high-resolution or high-frame-rate settings.

To enhance generalizability and robustness, alternative model architectures should be explored in future studies, and comparative evaluations should be conducted across models with different learning structures. Because only data from the same source were used for training and fine-tuning, expanding the dataset to include multicenter cases from various surgeons and surgical environments will be essential to minimize institutional or operator bias. Furthermore, establishing clinically meaningful cutoff values for bleeding areas is a crucial next step. Future studies analyzing broader clinical datasets are necessary to investigate how AI-derived quantitative metrics correlate with actual surgical outcomes (e.g., operative time and complication rates), thereby determining appropriate thresholds that can actively guide intraoperative hemostasis. Integrating multimodal information—such as irrigation flow rate, surgical instrument tracking, and intraoperative surgeon maneuvers—may further refine model performance and facilitate its practical application in real-time surgical workflows.

## 5. Conclusions

We developed and validated a deep learning model that can detect and quantify intraoperative bleeding during UBE procedures with high sensitivity and reproducibility. This model enables the objective assessment of visual field clarity from still images and surgical videos, offering a novel tool to support hemostatic technique evaluation, training, and quality monitoring in endoscopic spine surgery.

## Figures and Tables

**Table 1:** Diagnostic performance of individual surgeons compared to the consensus ground truth.

Evaluator	Accuracy	Sensitivity	Specificity	PPV	NPV
Surgeon 1	1.00	1.00	1.00	1.00	1.00
Surgeon 2	0.96	1.00	0.86	0.95	1.00
Surgeon 3	0.83	0.78	1.00	1.00	0.61
Surgeon 4	0.85	1.00	0.43	0.83	1.00
Surgeon 5	0.98	1.00	0.93	0.98	1.00
Average	0.92	0.96	0.84	0.95	0.92

**Table 2:** Per-image diagnostic performance of the best model for bleeding detection (180 GT masks; Wilson 95% CI).

Metric/Case	Value	95% CI
True Positive (TP)	129	–
True Negative (TN)	25	–
False Positive (FP)	17	–
False Negative (FN)	9	–
Accuracy	0.86	0.80–0.90
Sensitivity (Recall)	0.93	0.88–0.97
Specificity	0.60	0.44–0.73
Positive Predictive Value (PPV)	0.88	0.82–0.93
Negative Predictive Value (NPV)	0.74	0.57–0.85

**Table 3:** Dice and IoU of model inference masks for GT images by surgeons.

Group	Dice Median [IQR]	Dice Mean ± SD	IoU Median [IQR]	IoU Mean ± SD
Overall	0.79 [0.59–0.91]	0.67 ± 0.35	0.65 [0.42–0.84]	0.59 ± 0.34
Surgeon 1	0.81 [0.73–0.89]	0.74 ± 0.23	0.68 [0.57–0.79]	0.63 ± 0.24
Surgeon 2	0.76 [0.63–0.88]	0.67 ± 0.29	0.62 [0.46–0.77]	0.57 ± 0.29
Surgeon 5	0.65 [0.44–0.89]	0.57 ± 0.32	0.48 [0.26–0.71]	0.47 ± 0.31

IQR, interquartile range; SD, standard deviation.

**Table 4:** Dice and IoU analyses based on the GT agreement rates.

GT Agreement	N	Dice Median [IQR]	Dice Mean ± SD	IoU Median [IQR]	IoU Mean ± SD
≥0.70	34	0.71 [0.24]	0.72 ± 0.20	0.55 [0.31]	0.59 ± 0.23
≥0.80	23	0.81 [0.19]	0.80 ± 0.14	0.68 [0.26]	0.68 ± 0.19
≥0.90	14	0.89 [0.21]	0.84 ± 0.14	0.80 [0.31]	0.75 ± 0.20

IQR, interquartile range; SD, standard deviation.

**Table 5:** Dice and IoU analyses based on the GT bleeding area rates.

GT Bleeding Area Rate	N	Dice Median [IQR]	Dice Mean ± SD	IoU Median [IQR]	IoU Mean ± SD
Low bleeding (>0–20% of GT area)	56	0.53 [0.06–0.74]	0.45 ± 0.32	0.36 [0.03–0.59]	0.34 ± 0.27
High bleeding (>20% of GT area)	82	0.83 [0.73–0.91]	0.81 ± 0.15	0.71 [0.57–0.84]	0.70 ± 0.19

## Data Availability

The datasets generated during and/or analyzed during the current study are not publicly available, but are available from the corresponding author on reasonable request.

## Supplementary Materials

The following supporting information can be downloaded at: https://www.mdpi.com/article/10.3390/jcm15051934/s1, Video S1: UBE surgical video evaluation using the deep learning model.

## Abbreviations

The following abbreviations are used in this manuscript:

UBE	unilateral biportal endoscopic spine surgery
HSV	hue, saturation, and value
IoU	intersection-over-union
AI	artificial intelligence
RGB	red-green-blue
GT	ground-truth
PPV	positive predictive value
NPV	negative predictive value
CIs	Confidence intervals
